# Social learning may lead to population level conformity without individual level frequency bias

**DOI:** 10.1038/s41598-017-17826-9

**Published:** 2017-12-11

**Authors:** Kimmo Eriksson, Daniel Cownden, Pontus Strimling

**Affiliations:** 1 Stockholm University, Centre for the Study of Cultural Evolution, Stockholm, 106 91 Sweden; 2 Mälardalen University, School of Education, Culture & Communication, Västerås, 721 23 Sweden; 3 INgrooves, Victoria, BC, V8W 1T4 Canada; 4 Institute for Futures Studies, Stockholm, 101 31 Sweden

## Abstract

A requirement of culture, whether animal or human, is some degree of conformity of behavior within populations. Researchers of gene-culture coevolution have suggested that population level conformity may result from frequency-biased social learning: individuals sampling multiple role models and preferentially adopting the majority behavior in the sample. When learning from a single role model, frequency-bias is not possible. We show why a population-level trend, either conformist or anticonformist, may nonetheless be almost inevitable in a population of individuals that learn through social enhancement, that is, using observations of others’ behavior to update their own probability of using a behavior in the future. The exact specification of individuals’ updating rule determines the direction of the trend. These results offer a new interpretation of previous findings from simulations of social enhancement in combination with reinforcement learning, and demonstrate how results of dynamical models may strongly depend on seemingly innocuous choices of model specifications, and how important it is to obtain empirical data on which to base such choices.

## Introduction

Researchers of culture, whether human or animal, face the problem of how cultural differences between groups can persist over time despite processes like migration and intermarriage, which tend to mix individuals from different groups^1^. To sustain cultural differences between groups under such exchange of individuals requires some counteracting cultural force that promotes population level conformity. It may be tempting to think that social learning acts as such a force. However, consideration of a very simple model shows that social learning does not necessarily promote population level conformity^1,2^. Namely, assume two alternative behaviors, B and b, and assume that every learning individual independently draws a random role model from the population and copies the behavior of that role model. If the relative frequency of Bs among the role models is *f*, then the expected frequency of Bs among the learners is also *f*. Thus, social learning in this model does not amplify a difference in frequency between the two behaviors.

Robert Boyd and colleagues have shown how conformity can arise from social learning if individuals instead make *multiple observations and use some frequency bias* when learning from these observations^1,2^. For instance, an individual may observe a random sample of three members of the population and adopt the majority behavior in this sample^2^. With the same notation as above, the probability that an individual observes three Bs is *f* ^3^ while the probability of observing two Bs and one b is 3*f* ^2^ · (1 −*f* ). The probability of observing a majority of *B*s is the sum of these two terms, which can be rewritten as *f* + *f* · (1 − *f* ) · (2*f* − 1). The second term is positive when 1/2 < *f* < 1, which means that the frequency of the most common behavior will be greater among learners than among role models. In other words, this frequency-biased social learning model results in a conformist trend in the population.

Due to the abovementioned results, much theoretical research has focused on frequency-biased social learning as the mechanism behind sustained cultural differences between groups^1–4^. The empirical support that social learning is generally frequency-biased in this sense is mixed^5–14^. Based on simulations it has also recently been suggested that other individual biases in social learning, such as learning mainly from a smaller group of favored demonstrators, may produce the same kind of population-level trends as frequency-biased social learning^15^. However, follow-up research has argued that these findings were artifacts of the simulations^16^.

In a recent paper on animal traditions, Franz and Matthews suggested another way in which social learning could promote population level conformity in the absence of frequency bias, namely, if *individual behavior exhibits some randomness* and social learning interacts with reinforcement learning^17^. Franz and Matthews developed a simulation of population dynamics proceeding in discrete time steps. The state of an individual consisted of their probabilities for using either of two behaviors (say, B or b) as well as their level of knowledge for each behavior. At every time step, each individual displayed a behavior according to their current probabilities, received payoff based on their knowledge for the displayed behavior, updated their knowledge for that behavior, and updated the probability of using it in the next step based on the received payoff (i.e., reinforcement learning). These simulations did not bring about conformist population level trends. In another set of simulations, each time step was extended by letting each individual select another individual at random, observe the behavior of that individual, and *increase the probability of using the observed behavior*–a social learning mechanism referred to as *social enhancement*. The simulations that incorporated social enhancement exhibited conformist population level trends.

Note that, as social enhancement was based on a single observation, there was no frequency bias involved. Thus, the result of Franz and Matthews indicates that there are conditions under which social learning from randomly drawn role models can promote population level conformity even in the absence of frequency bias. The purpose of our paper is to examine what these conditions are.

One possibility was suggested by Franz and Matthews: it could be the combination of social enhancement with reinforcement learning that promotes conformity at the population level. Specifically, they suggested that social enhancement would amplify frequency differences arising from reinforcement^17^, p. 3368. However, this cannot hold in general. To see why, note that social enhancement includes as a special case the simple social learning model we discussed in the first paragraph. Namely, if social enhancement works by increasing your probability of using the observed behavior to 100%, it is equivalent to copying the observed behavior. And we know from the first paragraph that copying the observed behavior of a randomly drawn role model does *not* amplify frequency differences between behaviors.

In their simulations, Franz and Matthews used a specification of social enhancement in which probabilities were not increased all the way up to 100%. But there are infinitely many different ways in which one could specify the size of the increase. When discussing the population level effects of social enhancement we therefore need to take into account *exactly how large the increase in the probability of using the observed behavior is specified to be*. We shall demonstrate that, depending on the choice of specification, social enhancement may either promote conformity, or be neutral, or even promote anticonformity at the population level. Moreover, this holds regardless of whether or not social enhancement is combined with reinforcement learning. Thus, we aim to show that social enhancement is such a powerful mechanism that it does not require frequency bias, nor the presence of reinforcement learning, to cause population level trends.

Importantly, the domain of social enhancement is much larger if the presence of reinforcement learning is not required. Namely, social enhancement may then apply also to choices of behavior that are mainly socially determined, which is the focus of the models of cultural differences discussed in the initial paragraphs.

To gain detailed understanding of how social enhancement may lead to conformist or anticonformist population level trends, we study the effect at the population level of using different specifications of the probability increase function. For completeness we treat two different population level processes that have received attention in previous research on cultural evolution: between-generation learning and within-generation learning. Although they operate on different time-scales, these processes are quite similar from a mathematical perspective; we therefore expected the effects of varying the specification of social enhancement to be the same for between-generation and within-generation learning. Indeed, this is what we found.

We also discuss the effect on the individual of repeated social enhancement. We then turn to the combination of social enhancement with reinforcement learning. Finally, we look at the combination of social enhancement with frequency-biased processing of multiple observations.

## Results

We shall consider individuals who do not have detailed memory of all observations they have made in their lifetime. Instead of remembering the exact number of times they have observed *B* and the exact number of times they have observed b, they sacrifice some of this information by maintaining only a one-dimensional state, represented by their current probability *x* of doing B. This state variable is updated when a new observation of a B or a b is made. Because of this loss of historical information, the influence of any given observation may depend on when it is made. In other words, order effects may occur (as we will see in detail below).

Formally, we assume that an individual who observes behavior B at time *t* increases their own probability of using B from some current value *x*_*t*_ to a new value *x*_*t*+1_ according to some function incr(*x*_*t*_):

1 $${\rm{Observing}}\,{\rm{B}}\,{\rm{yields}}\,{x}_{t+1}={x}_{t}+{\rm{incr}}({x}_{t}).$$

As we assume a choice between only two behaviors, B and b, the probability of using the alternative behavior b at any time *t* is 1 − *x*_*t*_. If instead b is observed, we can just replace *x*_*t*_ by 1 − *x*_*t*_ in Eq. 1. After simplification we obtain the following:

2 $${\rm{Observing}}\,{\rm{b}}\,{\rm{yields}}\,{x}_{t+1}={x}_{t}-{\rm{incr}}(1-{x}_{t})\mathrm{.}$$

We shall consider various ways of specifying the function incr(*x*). As probabilities can only be in the range from 0 to 1, any specification must satisfy incr(*x*) ≤1 −*x*.

The exact specification of social enhancement that Franz and Matthews used can be found in the supplementary material to their paper. In our notation, their specification was

3 $${{\rm{incr}}}_{C}(x)={\gamma }_{C}\cdot \mathrm{(1}-x)\cdot x,{\rm{for}}\,{\rm{some}}\,{\rm{constant}}\,0\, < \,{\gamma }_{C}\le 1.$$

The index “C” is a label that we use to indicate that this particular specification leads to conformity at the population level, as we shall see below. Note that when the current behavioral probability *x* is close to zero, the value of incr_*C*_(*x*) is very small. In other words, if the individual is already almost always choosing b, a new observation of B will have almost no impact. This property can be regarded as akin to confirmation bias: the individual has become committed to one behavior and does not care if other people behave differently.

Other psychological theories may suggest other forms incr(*x*). Specifically we shall consider social impact theory, which states as a “law” that as the number of sources of social impact grows, the marginal impact decreases^18^. For instance, the greatest difference in impact should occur in the step from zero sources to one source. An interpretation of that law is that when an individual has previously only observed one type of behavior, say b, so that *x* has become close to zero, a first observation of B should have a large impact and subsequent observations of *B* should have less and less additional impact. We shall formulate two specifications of incr(*x*) that implements this principle. The first specification is

4 $${{\rm{incr}}}_{N}(x)={\gamma }_{N}\cdot \mathrm{(1}-x),{\rm{for}}\,{\rm{some}}\,{\rm{constant}}\,0\, < \,{\gamma }_{N}\le 1.$$

A good reason to consider this particular specification is that the special case of *γ*_*N*_ = 1 amounts to simply adopting the last observed behavior. The label “N” is used to indicate that, as we shall see, this specification leads to neutral drift at the population level. Note that the value of incr_*N*_(*x*) is proportional 1 − *x*. Thus, consistent with social impact theory, the impact of observing a B is at its largest when the current probability *x* of using B is small. Here, the impact of observing B decreases linearly with *x*. Finally, we consider a more extreme version where the impact of observing B decreases faster than linearly:

5 $${{\rm{incr}}}_{A}(x)={\gamma }_{A}\cdot {\mathrm{(1}-x)}^{2},{\rm{for}}\,{\rm{some}}\,{\rm{constant}}\,0\, < \,{\gamma }_{A}\le 1.$$

The label “A” is used to indicate that, as we shall see, this specification leads to anticonformity at the population level.

Below we present a framework for the analysis of the population level effects of a given specification of social enhancement. We then present the results of such analyses of the specifications incr_*C*_, incr_*N*_, and incr_*A*_. Thereafter, following Franz and Matthews, we use simulations to examine the interaction of social enhancement with reinforcement learning. Finally, we consider social enhancement combined with frequency biased processing of multiple observations.

### Analytical framework

We shall consider a large population of learning individuals, all using the same social enhancement mechanism to update the probabilities of using B vs. b.

In the between-generation analysis, we assume that each learning individual repeatedly observes an independently chosen random individual from a parent population. We assume that both behaviors are present in the parent population at some fixed frequencies. One behavior, say B, is assumed to be more common than the other. Thus, the parent population displays behavior B at a fixed frequency *f* satisfying 1/2 < *f* < 1. Then *f* is also the probability for an individual in the learning population to observe B at any given observation. To emphasize that the process is stochastic, we use capital letters to signify stochastic variables. The stochastic variable *X*_*t*_ denotes the probability that a given individual will use behavior B at time *t*. By the law of large numbers, the average frequency of behavior B in the learning population at time *t* will be very close to the expected value *E*[*X*_*t*_]. We say that a mechanism is *between-generation conformist-biased* if *E*[*X*_*τ*_] > *f* whenever *τ* is sufficiently large; *between-generation neutral* if *E*[*X*_*τ*_] is arbitrarily close to *f* whenever *τ* is sufficiently large; and *between-generation anticonformist-biased* if *E*[*X*_*τ*_] < *f* whenever *τ* is sufficiently large.

In the within-generation analysis, we assume a population that is learning from itself. In each time step, each individual both displays a behavior and observes the behavior of an independently chosen random individual in the same population. We say that the social enhancement mechanism is *within-generation conformist-biased* if the population trend will be toward homogeneity in displayed behavior (i.e., moving toward either all B or all b); *within-generation neutral* if the expected effect at the population level is zero; and *within-generation anticonformist-biased* if the population trend will be toward equal frequencies in display of the two behaviors.

### Population level analysis

For any *x* ∈ [0, 1], let *p*_*t*_(*x*) denote the probability that *X*_*t*_ = *x* under the process we are studying. Let *M*_*t*_ denote the finite set of values that *X*_*t*_ may in fact attain under that process:

6 $${M}_{t}\,:=\{x\in [0,1]:{p}_{t}(x) > 0\}$$

Probability theory then tells us that

7 $$\sum _{x\in {M}_{t}}{p}_{t}(x)=1,\quad \sum _{x\in {M}_{t}}x\cdot {p}_{t}(x)=E[{X}_{t}]={\overline{X}}_{t},{\rm{and}}\sum _{x\in {M}_{t}}{x}^{2}\cdot {p}_{t}(x)=E[{X}_{t}^{2}]\in [{\overline{X}}_{t}^{2},{\overline{X}}_{t}].$$

In the case of between-generation learning, a learning individual observes a B with probability *f*, leading to an increase in the individual’s own probability of using B. This increase is given by *X*_*t*+1_ − *X*_*t*_ = incr(*X*_*t*_). A b is observed with probability 1 − *f*, in which case the individual’s probability of using B is instead decreased according to *X*_*t*+1_ − *X*_*t*_ =− incr(1 − *X*_*t*_). It follows that the change in expected value is described by the equation

8 $${\overline{X}}_{t+1}-{\overline{X}}_{t}=E[{X}_{t+1}-{X}_{t}]=\sum _{x\in {M}_{t}}[{\rm{incr}}(x)\cdot f-{\rm{incr}}(1-x)\cdot (1-f)]\cdot {p}_{t}(x).$$

In the case of within-generation learning, the only difference is that a learning individual observes a B with probability equal to the frequency of display of B in the learning generation, which by the law of large numbers is very close to ${\overline{X}}_{t}$ in a large population. Thus, to conduct the analysis we just replace *f* by ${\overline{X}}_{t}$ in the above equation.

We shall now analyze between-generation learning and within-generation learning for each of three specifications of the function incr(*x*).

### Social enhancement by incr_*N*_ (*x*) is both between-generation and within-generation neutral

Using the specification incr_*N*_(*x*) = *γ*_*N*_ · (1 − *x*), Eq. 8 simplifies to

9 $$\begin{array}{ccc}{\overline{X}}_{t+1}-{\overline{X}}_{t} & = & {\gamma }_{N}\cdot \sum _{x\in {M}_{t}}(f-x)\cdot {p}_{t}(x)\end{array}$$

10 $$\begin{array}{ccc} & = & {\gamma }_{N}\cdot (f-{\overline{X}}_{t}),\end{array}$$

which can be rewritten as

11 $$f-{\overline{X}}_{t+1}=(1-{\gamma }_{N})\cdot (f-{\overline{X}}_{t}).$$

As 0 < *γ*_*N*_ ≤ 1, it follows that the sequence $f-{\overline{X}}_{t},\,f-{\overline{X}}_{t+1},\ldots$ will decrease and tend to zero as *t* grows. Thus, ${\overline{X}}_{\tau }$ will be arbitrarily close to *f* for sufficiently large *t*. Hence, incr_*N*_ is between-generation neutral according to our framework.

We now turn to within-generation learning for the same specification. Replacing *f* by ${\overline{X}}_{t}$ in Eq. 9 yields

12 $${\overline{X}}_{t+1}-{\overline{X}}_{t}=0.$$

This means that the expected change of behavior at the population level is zero (i.e., population level behavior will exhibit neutral drift). Hence, incr_*N*_ is within-generation neutral according to our framework.

### Social enhancement by incr_*C*_(*x*) is both between-generation and within-generation conformist-biased

Using the specification incr_*C*_(*x*) = *γ*_*C*_ · (1 − *x*) · *x*, Eq. 8 simplifies to

13 $${\overline{X}}_{t+1}-{\overline{X}}_{t}={\gamma }_{C}\cdot \sum _{x\in {M}_{t}}(2f-1)\cdot (1-x)\cdot x\cdot {p}_{t}(x),$$

which can be rewritten as

14 $$1-{\overline{X}}_{t+1}=E[(1-{X}_{t})\cdot (1-{\gamma }_{C}\cdot (2f-1)\cdot {X}_{t})].$$

As 0 < *γ*_*C*_ ≤ 1 and 1/2 < *f* < 1 it follows that the factor 1 − *γ*_*C*_ · (2*f* − 1) · *X*_*t*_ is strictly between zero and one, and hence the sequence $1-{\overline{X}}_{t},\,1-{\overline{X}}_{t+1},\ldots$ will decrease and tend to zero as *t* grows. This implies that ${\overline{X}}_{\tau } > f$ for all sufficiently large *τ*. Hence, incr_*C*_ is between-generation conformist-biased.

We now turn to within-generation learning for the same specification. Replacing *f* by ${\overline{X}}_{t}$ in Eq. 14 yields

15 $$1-{\overline{X}}_{t+1}=E[(1-{X}_{t})\cdot (1-{\gamma }_{C}\cdot (2{\overline{X}}_{t}-1)\cdot {X}_{t})].$$

We consider two cases. First assume that $1/2 < {\overline{X}}_{t} < 1$. As 0 < *γ*_*C*_ ≤ 1, it then follows that the factor $1-{\gamma }_{C}\cdot (2{\overline{X}}_{t}-1)\cdot {X}_{t}$ is strictly between zero and one, and hence the sequence $1-{\overline{X}}_{t},1-{\overline{X}}_{t+1},\ldots$ will decrease and tend to zero as *t* grows. Hence, ${\overline{X}}_{\tau }$ will be arbitrarily close to 1 for sufficiently large *τ*. In other words, the population will in this case trend toward homogeneous display of B.

Now consider the case $0 < {\overline{X}}_{t} < 1/2$ instead. We rewrite Eq. 15 as

16 $${\overline{X}}_{t+1}=E[{X}_{t}\cdot (1-{\gamma }_{C}\cdot (1-2{\overline{X}}_{t})\cdot (1-{X}_{t}))].$$

As 0 < *γ*_*C*_ ≤ 1 and $0\, < \,{\overline{X}}_{t}\mathrm{ < 1/2}$, the factor $1-{\gamma }_{C}\cdot \mathrm{(1}-2{\overline{X}}_{t})\cdot \mathrm{(1}-{X}_{t})$ is strictly between zero and one, and hence the sequence ${\overline{X}}_{t},{\overline{X}}_{t+1},\ldots$ will decrease and tend to zero as *t* grows, that is, ${\overline{X}}_{\tau }$ will be arbitrarily close to 0 for sufficiently large *τ*. In other words, the population will in this case trend toward homogeneous display of b. In sum, incr_*C*_ is within-generation conformist-biased.

### Social enhancement by incr_*A*_(*x*) is both between-generation and within-generation anticonformist-biased

Using the specification incr_*A*_(*x*) = *γ*_*A*_ · (1 − *x*)^2^, Eq. 8 simplifies to

17 $$\begin{array}{ccc}{\overline{X}}_{t+1}-{\overline{X}}_{t} & = & {\gamma }_{A}\sum _{x\in {M}_{t}}(f-2f\cdot x+(2f-1)\cdot {x}^{2})\cdot {p}_{t}(x)\end{array}$$

18 $$\begin{array}{ccc} & = & {\gamma }_{A}\cdot (f-{\overline{X}}_{t}+(1-2f)\cdot E[{X}_{t}\cdot (1-{X}_{t})]).\end{array}$$

For $\mathrm{1/2} < f\le {\overline{X}}_{t}$ this expression is negative, that is, the sequence ${\overline{X}}_{t},{\overline{X}}_{t+1},\ldots$ will decrease until the values are smaller than *f*. Hence, for all sufficiently large *τ* we must have ${\overline{X}}_{\tau } < f$, so incr_*A*_ is between-generation anticonformist-biased.

We now turn to within-generation learning for the same specification. Replacing *f* by ${\overline{X}}_{t}$ in Eq. 13 yields

19 $${\overline{X}}_{t+1}-{\overline{X}}_{t}={\gamma }_{A}\cdot (1-2{\overline{X}}_{t})\cdot E[{X}_{t}\cdot (1-{X}_{t})]).$$

This expression is positive whenever ${\overline{X}}_{t} < \mathrm{1/2}$ and negative whenever ${\overline{X}}_{t} > \mathrm{1/2}$. Hence, the change always brings ${\overline{X}}_{t+1}$ closer to 1/2, so that ${\overline{X}}_{\tau }$ will be arbitrarily close to 1/2 for all sufficiently large *τ*. According to our framework, incr_*A*_ is between-generation anticonformist-biased.

### Simulation of within-generation learning using varying specifications of social enhancement

**Figure 1:**
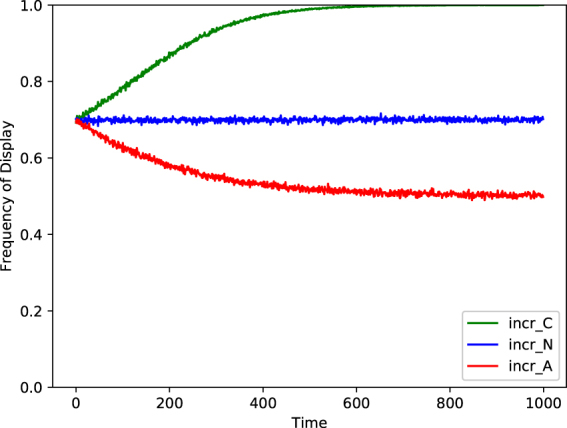
A plot of the proportion of behavior B displayed by a population of 1000 individuals as a function of time, for three simulated populations. Each simulation begins with an identical population, generated by drawing each individual’s value of *X*_*t*_ from a beta distribution with parameters *α* = 7 and *β* = 3. The blue (middle) curve is the trajectory followed by a population using social enhancement as specified by incr_*N*_, a within-generation neutral mechanism. The green (top) curve is the trajectory of a population using social enhancement as specified by incr_*C*_, a within-generation conformist-biased mechanism. The red (bottom) curve is the trajectory of a population using social enhancement as specified by incr_*A*_, a within-generation anticonformist-biased mechanism. We set *γ*_*N*_ = *γ*_*C*_ = *γ*_*A*_ = 0.01 in these simulations.

The above mathematical analysis of within-generation learning approximated frequencies of display with expected probabilities of display. This approximation is inaccurate for finite populations. To illustrate that the qualitative conclusions nonetheless hold, we simulated within-generation learning in a population of 1000 individuals. Over the course of *τ* = 1000 rounds, each of these individuals first made a display of behavior (B or b) according to the probability given by their current state variable, then observed a randomly chosen individual’s display, and finally updated their state probabilities according to the given specification of social enhancement. To clearly illustrate the differences between the population level trends caused by the three specifications of social enhancement, individual states were initialized so that the initial frequency of display of B in the population was approximately 0.7, Fig. 1 shows the results of the simulations: neutral drift when incr_*N*_ was used, a conformist trend when incr_*C*_ was used, and an anti-conformist trend when incr_*A*_ was used.

**Figure 2:**
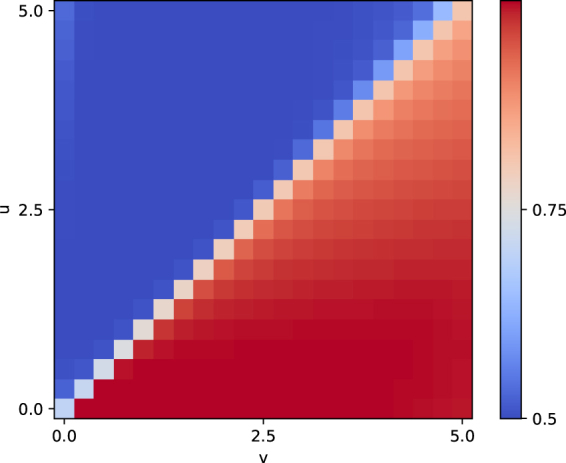
Each square in the grid represents the final frequency of display of behavior B (averaged over 5 simulations) for a given choice of *u* and *v* in the specification incr(*x*) = *γ* · (1 − *x*)^1+*u*^ · *x* ^*v*^. Values of *u* and *v* range between 0 and 5 in increments of 0.25. Note that above the diagonal, where *u* > *v* we consistently have final expected display frequencies close to 50% (i.e., anticonformist-bias); below the diagonal, where *u* < *v*, we consistently have average final expected display frequencies close to 100% (i.e., conformist-bias). In the lower left corner, where *u* = *v* = 0, the final expected display frequency is approximately the same as the initial display frequency of 70%. On the rest of the diagonal, where *u* = *v* > 0, the final expected display frequency is higher than 70% (i.e., indicating conformist-bias). Each simulation consists of 1000 individuals, run for 2000 rounds. The value of *γ* for each simulation is chosen so that the maximum possible change in an individual’s probability of display in a single round is 0.01.

The specifications we have studied have all been of the form incr(*x*) = *γ* · (1 − *x*)^1+*u*^ · *x* ^*v*^ for some values *u* ≥ 0 and *v* ≥ 0. To see how our analytic results on within-generation learning generalize across specifications of this form, we ran simulations with the same basic set up as described in the previous section but systematically varying the values of *u* and *v* in the range of [0.0, 5.0] in increments of 0.25 for a total of 441 distinct learning rules. See Fig. 2. Generalizing our analytic results, these simulations suggest that *u* > *v* results in anticonformist-biased mechanisms and that *u* < *v* results in conformist-biased mechanisms. Even when we set *u* = *v* > 0, the mechanism seemed to become slightly conformist-biased. These simulations illustrate that it may be very rare for a specification of social enhancement to be neutral. Rather, it seems that we should generally expect social enhancement to have a population level effect–either toward conformity or toward anticonformity.

### Individual level analysis

Note that population level trends arise despite individuals being unable to apply any frequency bias, as their social learning is based on a single observation at a time. Indeed, the population level trends in the three cases are determined by Eqs 12, 15 and 19, all of which describe the population level effect of individuals making a single observation.

One may wonder whether *repeated* social enhancement amounts to frequency bias at the individual level. In other words, does the frequency of Bs encountered during *τ* repetitions of social enhancement determine the individual’s final probability *x*_*τ*_ of using B? The answer is no, not even when the number *τ* of repetitions is very large.

To see this, consider an individual who starts out with *x*_0_ = 1/2 and updates this value following a series of observations of length *τ* in which the relative frequency of Bs is *f* > 1/2. The individual thus encounters *f* · *τ* Bs and (1 − *f*) · *τ* bs, where the number of Bs is greater than the number of bs. Consider two different orderings of these observations: Bs first (i.e., all Bs come before all bs), and Bs *last* (i.e., all bs come before all Bs). It turns out that for sufficiently large *τ* the following holds for the three specifications. (The proof is given in the supplementary material.)

- ${{\rm{incr}}}_{N}(x)$ In case of Bs last and sufficiently small *f* we have *x*_*τ*_ < *f*, but in case of Bs first we always have *x*_*τ*_ > *f*.
- ${{\rm{incr}}}_{N}(x)$ In case of Bs first we always have *x*_*τ*_ < *f*, but in case of Bs last we have *x*_*τ*_ > *f*.
- ${{\rm{incr}}}_{N}(x)$ Same as for ${{\rm{incr}}}_{N}(x)$.

Hence, at the individual level, none of these social enhancement mechanisms exhibits a well-defined sensitivity to the frequency of multiple observations. Only when averaged over the population is there a well-defined frequency sensitivity (as shown by the between-generation analyses above).

### Combining social enhancement with reinforcement learning

We now turn to the more elaborate simulation scheme used by Franz and Matthews, in which social enhancement is combined with reinforcement learning^17^. As described in the introduction, Franz and Matthews simulated a population of “individual learners” who only used reinforcement learning, as well as a population of “social enhancement learners” who added social enhancement to the reinforcement learning. Repeated simulations of the two populations (100 simulations of each population) produced systematically different outcomes with regards to conformity: In groups of individual learners, the mean difference in frequency between the more frequent behavior and the less frequent behavior at the end of the simulation was just 8 percentage points. Thus, reinforcement learning alone was insufficient for behavioral conformity to arise. In groups of social enhancement learners, however, the corresponding difference was 96 percentage points. Thus, the combination of reinforcement learning with social enhancement caused behavioral conformity.

In the introduction we outlined two competing explanations for this result: either conformity is produced by the interaction of reinforcement learning and social enhancement regardless of how you specify it, or it is the product of special properties of the specification of social enhancement that was used. In the preceding sections we have shown that, in the absence of reinforcement learning, social enhancement may produce both conformity and anticonformity depending on the specification. Franz and Matthews used the incr_*C*_(*x*) specification, which we now know produces conformity. To achieve a definitive test of the two explanations, we must examine the result of combining reinforcement learning with a specification of social enhancement that on its own would produce anticonformity.

We reran the exact same simulation of Franz and Matthews, only specifying social enhancement using incr_*A*_(*x*) instead. In groups of social learners, the mean difference in frequency between the more frequent behavior and the less frequent behavior at the end of the simulation was now less than 1 percentage point, that is, even lower than among individual learners.

Thus, the combination of reinforcement and social enhancement does not produce consistent outcomes. Our conclusion is that the original finding of emerging conformity obtained by Franz and Matthews reflects the special properties of the particular specification of social enhancement they used.

### Social enhancement based on multiple observations of behavior at a time

So far we have analyzed social enhancement based on a single observation at a time. If individuals instead make multiple observations at a time (or remember multiple recent observations), they may explicitly take frequency into account. How may such frequency bias in the processing of multiple observations interact with the bias we found to arise from different specifications of social enhancement?

There are many ways to model frequency bias and many ways to specify social enhancement. We shall address the question in one special case, building on a model of conformist social learning developed by Boyd and Richerson^2^. They modeled individuals that based their own behavior on the majority behavior observed among three random members of the population. Here we develop a social enhancement model that incorporates this particular frequency bias in the processing of multiple observations. Thus, we will assume that individuals observe the behavior of three random members of the population and use some specification of social enhancement to increase their probability of using the majority behavior observed among these three.

**Figure 3:**
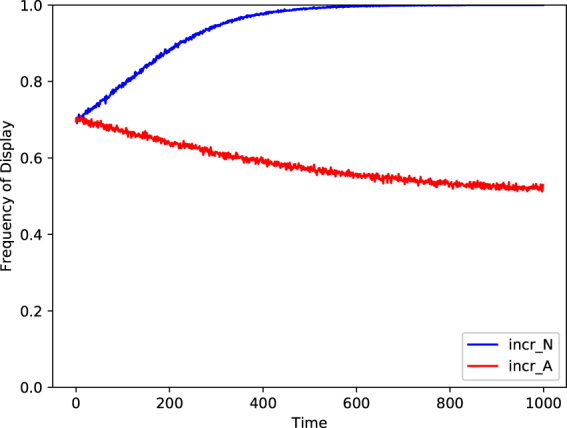
A plot of the proportion of the population displaying behavior B as a function of time for two simulated populations of 1000 individuals. Both simulations begin with an identical population, with each individual’s value of *X*_0_ drawn from a beta distribution with parameters *α* = 7 and *β* = 3. The blue (top) curve is the trajectory of a population using conformist-biased processing of observations combined with the incr_*N*_ specification of social enhancement (*γ*_*N*_ = 0.01). The red (bottom) curve is the trajectory of a population using conformist-biased processing of observations combined with the incr_*A*_ specification of social enhancement (*γ*_*A*_ = 0.01).

If the individual learner’s probability of using the majority behavior is increased to 100% (i.e., using specification incr_*N*_(*x*) with *γ*_*N*_ = 1), we are back in the model of Boyd and Richerson for which it is well-known that the population trend will be conformist. The interesting question is what the population trend will be if the individual learner’s probability of using the majority behavior is specified by incr_*A*_. In this case, a conformist bias in the processing of multiple observations is combined with an anticonformist bias in the specification of social enhancement. For this model of social enhancement we simulated within-generation learning in a population of 1000 individuals over 1000 time steps. The results are shown in Fig. 3. First, as expected, conformist-biased processing of observations combined with the incr_*N*_ specification of social enhancement yielded a conformist population trend. Second, and more interesting, conformist-biased processing of observations combined with the incr_*A*_(*x*) specification of social enhancement yielded an anticonformist population level trend.

We conclude that even in a population of individuals who are explicitly conformist-biased, certain specifications of social enhancement may still produce anticonformist cultural dynamics.

## Methods

Simulations were coded in Python using the NumPy package for scientific computing. The code for the simulations is available as Supplementary Information.

## Discussion

The overarching question behind this paper is how population level trends may arise from individual level social learning mechanisms. Much previous research has focused on how conformist population level trends may arise from individuals using some frequency bias when processing multiple observations.

Here we instead focused on how such trends may arise if individual behavior exhibits some randomness, with the probability of using a certain behavior updated whenever the individual observes a demonstrator using, or not using, that behavior. Thus, randomness in individual behavior is a key assumption. This assumption means that, even in the absence of additional social observations (or any other relevant cues to which behavior to use), the individual should sometimes vary its behavior from trial to trial. There are many possible sources of such randomness in behavior, such as a drive for exploration, a preference against monotony, influences of irrelevant stimuli or states, or otherwise noisy processing of the behavioral decision. It seems reasonable to assume that such factors will vary between species, between individuals, and between contexts.

An established framework for decision making distinguishes between two elements: a decision variable (DV) and a decision rule^19^. The DV is a state variable that is updated whenever new evidence is observed. When a decision is required, the decision rule states how the decision is made based on the value of the DV. In this framework, our model could be described as having a DV given by *x*_*t*_ and a probabilistic decision rule stating that behavior *B* should be used with probability *x*_*t*_. However, this constitutes a rather large leap from what DVs and decision rules usually look like in the decision-making literature, which is focused on situations where the world is in either of two states and where the individual’s choice should depend on its belief about the state of the world. The DV is then usually conceived of as the likelihood ratio (LR) that the world is in state 1 rather than in the alternative state 2^19^. In contrast, models of cultural evolution do not assume that cultural differences in behavior (e.g., style of expression in speech, art, clothes, etc.) hinge on different societies having different beliefs about the state of the world, but rather that these behaviors are shaped by individuals socially influencing each other. For that reason, we model social observations as acting directly on the individual’s probability of using one behavior over another.

Now recall our findings. In individual level analyses, we found that repeated social enhancement does not imply any well-defined frequency bias at the individual level. Nonetheless, population level analyses showed that repeated social enhancement may result in either conformist or anti-conformist population level trends, depending on the exact mathematical specification of social enhancement. This finding has several implications.

First, our results point to social enhancement as a hitherto unappreciated pathway by which population level trends can arise in humans and animals. Thereby, it adds to the debate on what inferences can be drawn from population-level data to individual-level mechanisms^15,16^.

A second implication is that predictions of population trends based on social enhancement is only possible if we obtain very detailed knowledge of how it works in practice at the individual level. In other words, we need to estimate, for a given individual in a given context, the mathematical form of the effect of observing a behavior on the probability that the observer will use that behavior. This is a very difficult task, not least because such probabilities are not directly observable.

Third, our results shed new light on a previous finding in simulations of animal behavior. Franz and Matthews found that population level conformity arose when they simulated a population of individuals that used a combination of reinforcement learning and social enhancement. They attributed this result to the interaction of these two mechanisms. Our analyses indicate that this conclusion was premature, and that the results obtained by Franz and Matthews do not rely on the presence of reinforcement, but strongly rely on the particular choice of mathematical specification of social enhancement that was used.

This example points to a final implication for modelers of cultural evolution. The setting up of models typically require arbitrary choices to be made, such as the choice of the exact specification social enhancement. Although such arbitrary choices may seem innocuous, our results demonstrate that they can have an overwhelming impact on the results. Without awareness of this possibility, it is likely that results of models are interpreted as holding in greater generality than they actually do.

## Electronic supplementary material

Supplementary information

## References

[1] Henrich J, Boyd R (1998). The evolution of conformist transmission and the emergence of between-group differences. Evol. Hum. Behav..

[2] Boyd, R. & Richerson, P. *Culture and the evolutionary process* (Univ. Chicago Pr. 1985).

[3] Henrich J, Boyd R (2001). Why people punish defectors: Weak conformist transmission can stabilize costly enforcement of norms in cooperative dilemmas. Journal of Theoretical Biology.

[4] Richerson, P. & Boyd, R. *Not by genes alone: How culture transformed human evolution* (Univ. Chicago Pr. 2005).

[5] Cialdini R, Goldstein N (2004). Social influence: Compliance and conformity. Annu. Rev. Psychol..

[6] Coultas JC (2004). When in rome… an evolutionary perspective on conformity. Group Processes & Intergroup Relations.

[7] Efferson C (2007). Learning, productivity, noise: An experimental study of cultural transmission on the Bolivian Altiplano. Evol. Hum. Behav..

[8] Efferson C, Lalive R, Richerson PJ, McElreath R, Lubell M (2008). Conformists and mavericks: The empirics of frequency-dependent cultural transmission. Evol. Hum. Behav..

[9] Eriksson K, Coultas J (2009). Are people really conformist-biased? an empirical test and a new mathematical model. J. Evol. Psychol..

[10] Eriksson K, Enquist M, Ghirlanda S (2007). Critical points in current theory of conformist social learning. J. Evol. Psychol..

[11] McElreath R (2005). Applying evolutionary models to the laboratory study of social learning. Evol. Hum. Behav..

[12] Morgan T, Rendell L, Ehn M, Hoppitt W, Laland K (2012). The evolutionary basis of human social learning. Proceedings of the Royal Society of London B: Biological Sciences.

[13] Morgan TJ, Laland KN, Harris PL (2015). The development of adaptive conformity in young children: effects of uncertainty and consensus. Developmental Science.

[14] Muthukrishna M, Morgan TJ, Henrich J (2016). The when and who of social learning and conformist transmission. Evolution and Human Behavior.

[15] Acerbi, A., Van Leeuwen, E. J., Haun, D. B. & Tennie, C. Conformity cannot be identified based on population-level signatures. *Scientific reports* **6** (2016). 10.1038/srep36068 PMC5086853 27796373

[16] Smaldino, P. E., Aplin, L. M. & Farine, D. R. Do sigmoidal acquisition curves indicate conformity? *bioRxiv* 159038 (2017).

[17] Franz M, Matthews L (2010). Social enhancement can create adaptive, arbitrary and maladaptive cultural traditions. Proceedings of the Royal Society B: Biological Sciences.

[18] Latane B (1981). The psychology of social impact. American Psychologist.

[19] Gold JI, Shadlen MN (2007). The neural basis of decision making. Annu. Rev. Neurosci..

