# Supplementary material for: Social learning may lead to population level conformity without individual level frequency bias
Source: Sci Rep. 2017 Dec 11;7:17341. doi: 10.1038/s41598-017-17826-9 (PMC5725437; doi:10.1038/s41598-017-17826-9)
Supplement: Supplementary file 1 — Supplementary information [file 41598_2017_17826_MOESM1_ESM.pdf]

Supplementary material for ”Social learning may lead to population level conformity without individual level frequency bias” by Kimmo Eriksson, Daniel Cownden, and Pontus Strimling

Here we provide the proofs that, at the individual level, none of the social enhancement mechanisms exhibits a well-defined sensitivity to the frequency of multiple observations.

Proof for $\mathrm{incr}_C(x)$ and Bs last

Define f^* by

$$f^* = \begin{cases} 1 & \text{if } \gamma_C = 1, \\ \frac{\ln(1/(1-\gamma_C))}{\ln(1+\gamma_C)+\ln(1/(1-\gamma_C))} & \text{if } 0 < \gamma_C < 1. \end{cases} \quad (1)$$

It is straightforward to verify that the definition of f^* implies

$$(1-\gamma_C)^{(1-f^*)} \cdot (1+\gamma_C)^{f^*} = 1. \quad (2)$$

We analyze the effect of a series of observations with Bs last by treating the bs and the Bs separately. For the first $(1-f)\cdot\tau$ time steps the observed behavior is always b, yielding $x_{t+1} = (1-\gamma_C)\cdot x_t + \gamma_C \cdot x_t^2$, which quickly tends to zero. For any $\epsilon > 0$ and for sufficiently large t we therefore have $x_{t+1} < (1+\epsilon-\gamma_C)\cdot x_t$, and for sufficiently large τ we consequently have $x_{(1-f)\tau} < x_0 \cdot (1+\epsilon-\gamma_C)^{(1-f)\tau}$. The second stage consists of the remaining $f\cdot\tau$ time steps in which the observed behavior is always B, yielding $x_{t+1} = (1+\gamma_C)\cdot x_t - \gamma_C \cdot x_t^2 < (1+\gamma_C)\cdot x_t$ for all time steps in the second stage. Combining the two stages, we have for all sufficiently large τ that

$$x_\tau < x_0 \cdot (1+\epsilon-\gamma_C)^{(1-f)\tau} \cdot (1+\gamma_C)^{f\tau}. \quad (3)$$

The right-hand side expression is increasing in f and decreasing in ϵ. By continuity it then follows that for all $f < f^*$ we can choose an $\epsilon > 0$ such that

$$x_\tau < x_0 \cdot (1-\gamma_C)^{(1-f^*)\tau} \cdot (1+\gamma_C)^{f^*\tau}, \quad (4)$$

for all sufficiently large τ. By Eq. 2 the right-hand expression equals $x_0 \cdot 1^\tau = x_0 = 1/2 < f$. Hence, $x_\tau < 1/2 < f$ for all $f < f^*$ and all sufficiently large τ.

Proof for $\mathrm{incr}_C(x)$ and Bs first

To study the case of Bs first, we can mimic the proof for the previous case if we study $1 - x_t$ instead of x_t. Corresponding to Eq. 4 we then obtain

$$1 - x_\tau < (1-x_0) \cdot (1-\gamma_C)^{(f^*\tau} \cdot (1+\gamma_C)^{(1-f^*)\tau}, \quad (5)$$

for all sufficiently large τ. By Eq. 2 the right-hand expression equals

$$(1-x_0)\cdot(\frac{1-\gamma_C}{1+\gamma_C})^{(2f^*-1)\tau}, \tag{6}$$

which tends to zero when τ tends to infinity, as $f > 1/2$. Hence, Eq. 5 implies that x_τ tends to 1, and hence that $x_\tau > f$ for all sufficiently large τ.

Proof for $\mathrm{incr}_N(x)$ and Bs first

After the initial $f\cdot\tau$ observations of B we necessarily have $x_{f\tau} \leq 1$. Each of the $(1-f)\cdot\tau$ subsequent observations of b will then, according to specification incr_N, result in a decrease by a factor of $(1-\gamma_N)$. The final value therefore satisfies

$$x_\tau \leq (1-\gamma_N)^{(1-f)\cdot\tau}. \tag{7}$$

It follows that x_τ tends to zero when τ tends to infinity. Hence $x_\tau < f$ for all sufficiently large τ.

Proof for $\mathrm{incr}_N(x)$ and Bs last

To study the case of Bs last, we can mimic the proof for the previous case if we study $1-x_t$ instead of x_t. It follows that $1-x_\tau$ tends to zero when τ tends to infinity. Hence $x_\tau > f$ for all sufficiently large τ.

Proof for $\mathrm{incr}_A(x)$

The above proofs for the case of $\mathrm{incr} = \mathrm{incr}_N$ apply also to the case of $\mathrm{incr} = \mathrm{incr}_A$ after replacing $(1-\gamma_N)$ with $(1-\gamma_A)^2$.

Supplementary material for "Social learning may lead to population level conformity without individual level frequency bias" by Kimmo Eriksson, Daniel Cownden, and Pontus Strimling

Python code for the simulations

```
import numpy as np
import pdb
import matplotlib.pylab as pl
from matplotlib import cm

N = 10000 # population szie
T = 1000 # Length of simulation

def runSim (u=0.0,v=0.0, gamma=None, initP = None, earlyStop = False):
    freqHist = np.zeros(T) #history of frequency of display
    EfreqHist = np.zeros(T)
    if initP is None:
        P = np.random.beta(7,3,N) #initialize display probabilities from a beta distribution
    else:
        P = initP
    if gamma == None:
        maxStep = ((u+1.0)/(u+v+1.0))**(u+1.0) * ((v)/(u+v+1.0))**(v)
        gamma = 0.01 / maxStep

    for t in range(T):
        if max(P) > 1.0:
            pdb.set_trace()
        D = np.random.binomial(1,P)
        freqHist[t] = np.mean(D)
        EfreqHist[t] = np.mean(P)
        Obs = np.random.choice(D, size = N, replace=True)
        P = P +  gamma * (Obs * ((1-P)**(u+1.0) * P**v + P**(u+1.0) * (1-P)**v) - (P**(u+1) *
(1-P)**v))

        if np.any(np.isnan(P)):
            pdb.set_trace()

        if np.mean(P) < 0.4:
            pdb.set_trace()

        if earlyStop:
            if np.mean(P) > 0.9999:
                EfreqHist[-1] = 0.9999
                print('earlyStop: ' + str(t))
                break
    return freqHist, EfreqHist

def runObsSim (u=0.0,v=0.0,gamma=0.01, initP = None, num_obs=3):
    freqHist = np.zeros(T) #history of frequency of display
    EfreqHist = np.zeros(T)
    if initP is None:
        P = np.random.beta(7,3,N) #initialize display probabilities from a beta distribution
    else:
        P = initP

    for t in range(T):
        if max(P) > 1.0:
            pdb.set_trace()
        D = np.random.binomial(1,P)
        freqHist[t] = np.mean(D)
        EfreqHist[t] = np.mean(P)
        Obs = np.random.choice(D, size = (N,num_obs), replace = True)
        Obs = np.sum(Obs, 1)
        Obs = (Obs >= (num_obs/2.0)).astype(np.float32)
        P = P +  gamma * (Obs * ((1-P)**(u+1.0) * P**v + P**(u+1.0) * (1-P)**v) - (P**(u+1) *
(1-P)**v))
    return freqHist, EfreqHist
```

```
def runGridSim(uMax=5.0, vMax=5.0, s = 21, reps=5):
    uvals = np.linspace(0, uMax, s)
    vvals = np.linspace(0, vMax, s)

    results = np.zeros((len(uvals), len(vvals), reps))
    for ii,u in enumerate(uvals):
        for jj,v in enumerate(vvals):
            maxStep = ((u+1.0)/(u+v+1.0))**(u+1.0) * ((v)/(u+v+1.0))**(v)
            scale = 0.01 / maxStep
            print(maxStep)
            for rr in range(reps):
                print(str(u) + ', ' + str(v) + ', ' + str(rr))
                freqHist, EfreqHist = runSim(u=u, v=v, gamma=scale, earlyStop = True)
                results[ii,jj,rr] = EfreqHist[-1]
                meanResults = np.mean(results, 2)
    return results, meanResults

def makeFig1():
    fig = pl.figure(1)
    ax1 = fig.add_subplot(111)

    initP = np.random.beta(7,3,N) #initialize display probabilities from a beta distribution

    f_homo, ef_homo = runSim(u=0.0, v=1.0, gamma=0.01, initP=initP)
    ax1.plot(f_homo, linestyle='-', color='green', label = 'incr_C') #homogeneous, P(1-P)

    f_nue, ef_nue = runSim(u=0.0, v=0.0, gamma=0.01, initP=initP)
    ax1.plot(f_nue, linestyle='-', color='blue', label = 'incr_N') #neutral, (1-P)

    f_hetero, ef_hetero = runSim(1.0, 0.0, 0.01, initP)
    ax1.plot(f_hetero, linestyle='-', color='red', label = 'incr_A') #heterogeneous (1-P)^2

    ax1.set_ylabel('Frequency of Display')
    ax1.set_xlabel('Time')
    #ax1.set_title('Without Conformist Transmission Bias')
    ax1.set_ylim([0,1])
    ax1.legend(loc='lower right')
    #ax1.set_title('The population level effects of three learning Rules')
    pl.savefig('fig1.eps')
    #pl.show()

def makeFig2(result):
    fig = pl.figure(2)
    ax1 = fig.add_subplot(111)
    cax = ax1.imshow(result, interpolation='nearest', cmap=cm.coolwarm, origin = 'lower')
    cbar = fig.colorbar(cax, ticks=[0.5, 0.75, 1.0])
    cbar.ax.set_yticklabels(['0.5', '0.75', '1.0'])

    ax1.set_xlabel('v')
    ax1.set_xticks(np.linspace(0,20,3))
    ax1.set_xticklabels(np.linspace(0,5,3))

    ax1.set_ylabel('u')
    ax1.set_yticks(np.linspace(0,20,3))
    ax1.set_yticklabels(np.linspace(0,5,3))

    pl.savefig('fig2.eps')


def makeFig3():
    fig = pl.figure(3)
    ax1 = fig.add_subplot(111)

    initP = np.random.beta(7,3,N) #initialize display probabilities from a beta distribution

    f_nue, ef_nue = runObsSim(0.0, 0.0, 0.01, initP=initP, num_obs=3)
    ax1.plot(f_nue, linestyle='-', color='blue', label = 'incr_N') #neutral, (1-P)

    f_hetero, ef_hetero = runObsSim(1.0, 0.0, 0.01, initP)
    ax1.plot(f_hetero, linestyle='-', color='red', label = 'incr_A') #heterogeneous, (1-P)^2
```

```
ax1.set_ylabel('Frequency of Display')
ax1.set_xlabel('Time')
#ax1.set_title('With Conformist transmission Bias')
ax1.set_ylim([0,1])
ax1.legend(loc='lower right')
#ax1.set_title('The population level effects of three learning Rules')
#pl.show()
pl.savefig('fig3.eps')
```
